# Long-term outcomes of delayed clozapine initiation in treatment-resistant schizophrenia: a multicenter retrospective cohort study

**DOI:** 10.1186/s12888-023-05176-y

**Published:** 2023-09-15

**Authors:** Masakazu Hatano, Hiroyuki Kamei, Ippei Takeuchi, Kazuhiko Gomi, Takashi Sakakibara, Shogo Hotta, Satoru Esumi, Kiyotaka Tsubouchi, Yoshihito Shimizu, Shigeki Yamada

**Affiliations:** 1 Department of Pharmacotherapeutics and Informatics, Fujita Health University School of Medicine, 1-98 Dengakugakubo, Kutsukake, Toyoake, Aichi Japan; 2 Office of Clinical Pharmacy Practice and Health Care Management, Faculty of Pharmacy, Meijo University, Nagoya, Japan; 3 Department of Psychiatry, Okehazama Hospital, Toyoake, Japan; 4 Nagano Prefectural Mental Wellness Center Komagane, Komagane, Japan; 5 Department of Psychiatry, Holy Cross Hospital, Toki, Japan; 6 Department of Hospital Pharmacy, Nagoya University School of Medicine, Nagoya, Japan; 7 Division of Clinical Sciences and Neuropsychopharmacology, Graduate School of Pharmacy, Meijo University, Nagoya, Japan; 8 Department of Pharmacy, Okayama University Hospital, Okayama, Japan; 9 The Faculty of Pharmaceutical Sciences, Kobe Gakuin University, Kobe, Japan; 10 Department of Hospital Pharmacy, Kanazawa University, Kanazawa, Japan; 11 Department of Pharmacy, Kanazawa Medical University Hospital, Kahoku, Japan

**Keywords:** Antipsychotic agents, Time-to-treatment, Hospitalization, Early medical intervention, Secondary prevention

## Abstract

**Background:**

Clozapine is the only antipsychotic medication with proven efficacy against treatment-resistant schizophrenia. This multicenter retrospective cohort study aimed to evaluate the impact of a delay in clozapine initiation on long-term outcomes.

**Methods:**

Patients who initiated clozapine treatment between July 2009 and December 2018 were included in this study. According to the length of time from the diagnosis of schizophrenia to clozapine initiation, the patients were categorized into one of three groups: early (≤ 9 years), intermediate (10–19 years), and late (≥ 20 years) initiation. The endpoints were psychiatric rehospitalization and all-cause clozapine discontinuation within 3 years. Hazard ratios (HR) and 95% confidence interval (CI) were estimated using the Fine and Gray method or the Cox proportional hazards model.

**Results:**

The incidence rates of rehospitalization within three years, according to the cumulative incidence function, were 32.3% for early, 29.7% for intermediate, and 62.2% for late initiation, respectively. Late initiation had a significantly higher risk of psychiatric rehospitalization than early initiation (HR, 2.94; 95% CI, 1.01– 8.55; *P* = 0.016 by the Gray's test). The risk of psychiatric rehospitalization was not significantly different between the early and intermediate initiation groups. The incidence rate of all-cause clozapine discontinuation within three years using the Kaplan–Meier method was 13.0% for early, 10.6% for intermediate, and 20.1% for late initiation. The risk of all-cause clozapine discontinuation was not significantly among the groups. The late initiation group had more patients discontinuing because of death due to physical diseases than the other groups.

**Conclusions:**

The study suggests that clozapine should be initiated promptly in patients with treatment-resistant schizophrenia to prevent psychiatric rehospitalization during long-term treatment. Further prospective studies with appropriate consideration of confounding factors and large sample sizes are needed to strengthen the evidence.

## Background

Schizophrenia is a chronic mental disorder with a lifetime prevalence of 0.7% and is known to relapse repeatedly, even after remission [1]. Antipsychotics are the most effective medications for schizophrenia. However, approximately 30% of patients have treatment-resistant schizophrenia (TRS), which responds poorly to antipsychotic medications [2]. In Japan, a patient is diagnosed with TRS if he or she “never reached 41 points or more on the Global Assessment of Functioning” despite “at least two atypical antipsychotic medications” administered at a dose of “over 600 mg/d of chlorpromazine” for “over four weeks” [3].

Clozapine is the only antipsychotic medication with proven efficacy against TRS. Clozapine is the most effective antipsychotic medication in improving overall psychiatric symptoms and carries a low risk for akathisia, prolactin elevation, or the necessity of using anti-Parkinson medication [4]. In a population-based cohort study, clozapine was shown to carry the lowest risk for psychiatric rehospitalization and treatment failure (a composite outcome that includes psychiatric rehospitalization, discontinuation, and death) [5]. Despite the superior clinical outcomes reported for clozapine compared to other antipsychotics, it is still not widely used worldwide, especially in Japan, where it is rarely prescribed [6, 7]. In addition, clozapine initiation has been shown to have an average delay of 5 years from meeting the criteria for TRS [8], and this delay may have an important adverse impact on clinical outcomes. Delayed clozapine initiation is also correlated with a lower rate of psychiatric symptom improvement, and clozapine should be initiated within 3 years of the diagnosis of TRS [9, 10]. About 25% of TRS is clozapine-resistant (defined as a poor response to 6 weeks of a sufficient dose of clozapine treatment), and that delayed clozapine initiation is an important risk factor [11].

Most studies examining the impact of delayed clozapine initiation on clinical outcomes have analyzed its association with treatment response, i.e., various psychiatric rating scales (e.g., Positive and Negative Syndrome Scale and Brief Psychiatric Rating Scale) [12]. Rehospitalization and treatment discontinuation in long-term observations are also important measures of antipsychotic efficacy; however, few associations with these outcomes have been reported. The only study that examined the association between delayed clozapine initiation and hospitalization rates after clozapine initiation failed to reach statistical significance [13]. This study aimed to conduct a retrospective chart review and evaluate the impact of delayed clozapine initiation on rehospitalization and treatment discontinuation.

## Methods

### Study design

This was a multicenter retrospective cohort study based on patient clinical data from medical records. Clinical data were collected from eight sites in Japan (Fujita Health University Hospital, Nagoya University Hospital, Okayama University Hospital, Kanazawa University Hospital, Kanazawa Medical University Hospital, Okehazama Hospital, Nagano Prefectural Mental Wellness Center Komagane, and Holy Cross Hospital). We investigated patient characteristics (age at clozapine initiation, sex, history of modified electroconvulsive therapy, and time from the diagnosis of schizophrenia to the clozapine initiation) at the time of clozapine initiation, days from clozapine initiation to discharge, and following clinical course (psychiatric rehospitalization and discontinuation of clozapine). The observation period for this study was 3 years after discharge in hospitalization for clozapine initiation.

### Patients

Patients who were 20 years of age or older, met the diagnostic criteria for schizophrenia in the Diagnostic and Statistical Manual of Mental Disorders, Fourth, Fourth-text revision, or Fifth Edition, and were started on clozapine between July 2009 and December 2018 were included. Patients who were not discharged within the inclusion period or for whom clozapine was discontinued prior to discharge were excluded because this study evaluated long-term outcomes in the maintenance phase after discharge.

### Assessment

Patients were categorized into one of three groups based on the time from the diagnosis of schizophrenia to clozapine initiation: early (≤ 9 years), intermediate (10–19 years), and late (≥ 20 years) initiation. The primary outcome was psychiatric rehospitalization within 3 years. The secondary outcomes were all-cause clozapine discontinuation (including death) within 3 years. Other outcomes were reasons for discontinuation of clozapine.

### Statistical analysis

Comparisons of baseline patient characteristics were evaluated using analysis of variance for continuous variables and Fisher's exact test for nominal scales.

Psychiatric rehospitalization and all-cause clozapine discontinuation were analyzed with early initiation as the reference and the other two groups (intermediate and late initiation) as comparisons. We evaluated psychiatric rehospitalization during clozapine treatment in this study. Clozapine discontinuation (including death) is considered a competing risk because it precludes psychiatric rehospitalization events. Competing risk events may overestimate the primary event of interest using the usual Kaplan–Meier method. Therefore, the incidence rate of psychiatric rehospitalization was calculated using the cumulative incidence function that considered all-cause clozapine discontinuation as a competing risk [14]. The time to psychiatric rehospitalization was analyzed by Gray's test. Hazard ratios (HR) and 95% confidence interval (CI) were estimated using the Fine and Gray method of competing risk regression analysis. The incidence rate of all-cause clozapine discontinuation was calculated using the Kaplan–Meier method. Time to all-cause clozapine discontinuation was analyzed by the log-rank test. HRs and 95% CI were estimated using the Cox proportional hazards model. Both multivariate analyses were adjusted for four covariates (age at clozapine initiation, sex, number of days from clozapine initiation to discharge, and history of modified electroconvulsive therapy). The observation period for all time-to-event analyses was 3 years, and patients who could not be followed up were treated as censored.

The sample size was based on the number of subjects enrolled during the study period. All statistical analyses were performed using R 4.2.0 (The R Foundation for Statistical Computing, Vienna, Austria).

## Results

### Patient disposition and characteristics

The medical records of 135 patients who were initiated on clozapine during the study period were reviewed. After one patient with incomplete clinical information was excluded, 134 were analyzed for this study. Patient characteristics are summarized in Table 1.

**Table 1:** Baseline patient characteristics

	All (*n* = 134)	Early-initiation : ≤ 9 years	Intermediate-initiation : 10–19 years	Late-initiation : ≥ 20 years	*P*-value
		(*n* = 32)	(*n* = 51)	(*n* = 51)	
Age (y) at clozapine initiation, mean ± SD	41.6 ± 11.7	33.8 ± 10.8	37.1 ± 7.3	50.9 ± 9.6	< 0.001
Male sex (%)	69 (51.5)	18 (56.3)	27 (52.9)	24 (47.1)	0.70
Days from clozapine initiation to discharge, mean ± SD	257.5 ± 357.6	196.1 ± 238.8	250.8 ± 370.4	302.6 ± 403.9	0.42
History of modified electroconvulsive therapy (%)	14 (10.4)	5 (15.6)	5 (9.8)	4 (7.8)	0.51

*Abbreviations*: *SD* standard deviation

### Time from diagnosis to clozapine initiation

**Fig. 1:**
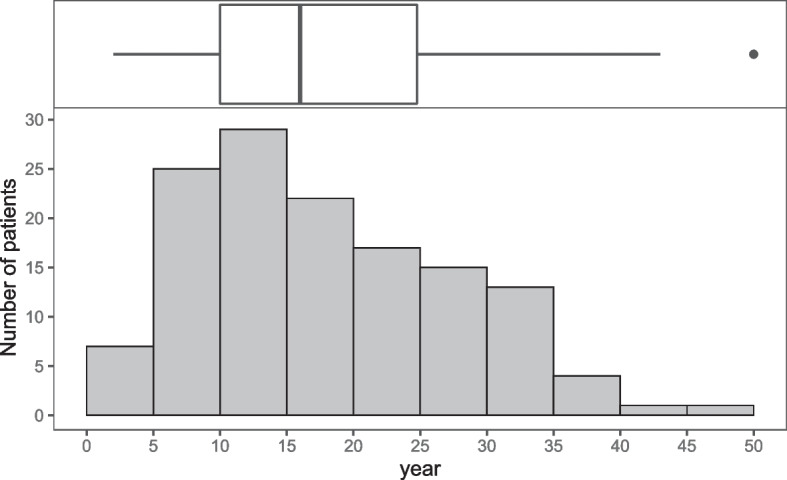
Time from diagnosis of schizophrenia to clozapine initiation

The median (interquartile range) time from diagnosis of schizophrenia to clozapine initiation was 16 years (10–25). Clozapine was initiated between 10 and 14 years after diagnosis of schizophrenia 29 (21.6%) patients, and within 4 years in only 7 (5.2%) patients; time from diagnosis to clozapine initiation are shown in Fig. 1. Early (≤ 9 years), intermediate (10–19 years), and late (≥ 20 years) initiation were 23.9% (32 patients), 38.1% (51 patients), and 38.1% (51 patients), respectively (Table 1). Age at clozapine initiation was significantly different among the three groups.

### Time-to-event analysis

**Fig. 2:**
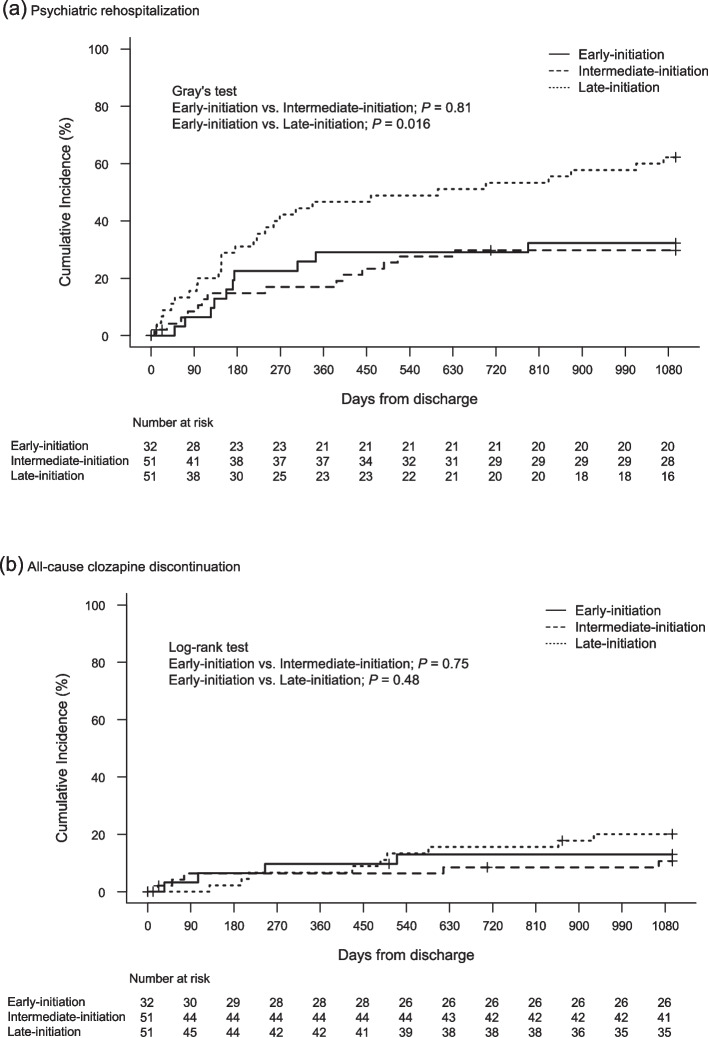
Time to psychiatric rehospitalization and all-cause clozapine discontinuation

The results of the time-to-event analysis for each endpoint are summarized in Table 2. The incidence rates of psychiatric rehospitalization within 3 years according to the cumulative incidence function were 32.3% for early, 29.7% for intermediate, and 62.2% for late initiation, respectively (Fig. 2a). Late initiation had a significantly higher risk of psychiatric rehospitalization than early initiation (HR, 2.94; 95% CI, 1.01– 8.55; *P* = 0.016 by the Gray's test). The risk of psychiatric rehospitalization was not significantly different between the early and intermediate initiation groups.

**Table 2:** Hazard rations for primary and secondary outcomes

	Psychiatric rehospitalization		All-cause clozapine discontinuation	
	Unadjusted HR (95% CI)	Adjusted HR (95% CI)	Unadjusted HR (95% CI)	Adjusted HR (95% CI)
Early vs. Intermediate	0.90 (0.40–2.05)	0.94 (0.40–2.21)	0.80 (0.21–2.98)	0.78 (0.20–3.02)
Early vs. Late initiation	2.36 (1.15–4.84)	2.94 (1.01–8.54)	1.54 (0.47–4.99)	0.44 (0.11–1.76)
Age at clozapine initiation	-	0.99 (0.95–1.03)	-	1.08 (1.03–1.13)
Male sex	-	0.94 (0.54–1.62)	-	0.73 (0.26–2.00)
Days from clozapine initiation to discharge	-	1.00 (1.00–1.00)	-	1.00 (1.00–1.00)
History of modified electroconvulsive therapy	-	1.46 (0.59–3.65)	-	1.57 (0.43–5.70)

*Abbreviations*: *HR* hazard ratio, *CI* confidence interval

The incidence rates of all-cause clozapine discontinuation within 3 years using the Kaplan–Meier method were 13.0% for early, 10.6% for intermediate, and 20.1% for late initiation (Fig. 2b). The risk of all-cause clozapine discontinuation was not significantly among the groups. After adjustment for covariates, only age at clozapine initiation was significantly associated with all-cause clozapine discontinuation (HR, 1.08; 95% CI, 1.03–1.13).

### Reasons for clozapine discontinuation

The reasons for discontinuing clozapine are summarized in Table 3. Eighteen patients discontinued clozapine during the 3-year observation period (4, 5, and 9 in the early, intermediate, and late initiation groups, respectively). The main reasons cited for discontinuation were lack of efficacy, death due to physical diseases, poor adherence to medication, and patient’s/family’s wishes in 3.0% (4 patients) each. The late initiation group had more patients discontinuing because of death due to physical diseases than the other groups.

**Table 3:** Reasons for clozapine discontinuation

	All (*n* = 134)	Early initiation (≤ 9 years)	Intermediate initiation (10–19 years)	Late initiation (≥ 20 years)
		(*n* = 32)	(*n* = 51)	(*n* = 51)
Lack of efficacy	4 (3.0)	1 (3.1)	1 (2.0)	2 (3.9)
Death due to physical diseases	4 (3.0)	1 (3.1)	0 (0.0)	3 (5.9)
Poor medication adherence	4 (3.0)	0 (0.0)	2 (3.9)	2 (3.9)
Patient or family wishes	4 (3.0)	1 (3.1)	1 (2.0)	2 (3.9)
Treatment of physical disease	1 (0.7)	1 (3.1)	0 (0.0)	0 (0.0)
Neutropenia	1 (0.7)	0 (0.0)	1 (2.0)	0 (0.0)
Fever	1 (0.7)	0 (0.0)	0 (0.0)	1 (2.0)

Patients who discontinued clozapine due to multiple factors were counted for each reason

## Discussion

The impact of delayed clozapine initiation on clinical outcomes indicate that early intervention with clozapine is beneficial. We evaluated the association between delayed clozapine initiation and long-term outcomes (psychiatric rehospitalization and all-cause clozapine discontinuation) over 3 years. The results of this study revealed that patients with late initiation had a significantly higher risk of psychiatric rehospitalization than the other two groups of patients. Thus, early intervention with clozapine is useful in improving psychiatric symptoms and preventing relapse.

All-cause clozapine discontinuation was not significantly associated with delayed clozapine initiation, only with the age at which clozapine was initiated. A national database study in Chile reported that an older age at clozapine initiation and delayed clozapine initiation are associated with clozapine discontinuation [15]; our results are partly consistent. Because clozapine is considered the last treatment option, the probability of switching to other antipsychotics is low. The low discontinuation rate of clozapine (10–20% in our study) may have been falsely negative due to low statistical power. In addition, because death was included in all-cause clozapine discontinuation, the influence of age-related physical disease is a confounder.

Studies differ in their definitions of delayed clozapine initiation. According to a systematic review of the impact of delayed clozapine initiation, the most common method defines it as the time from diagnosis of TRS (i.e., at the time of poor response to second non-clozapine antipsychotic) to clozapine initiation [12]. We considered the same approach, but obtaining an accurate medication history was difficult and we were unable to adopt this definition. Therefore, we used the time from schizophrenia diagnosis to clozapine initiation as an alternative measure, similar to the method used by Harrison et al. (2010) [13]. Two cohort studies reported that 70–80% of patients with TRS are already treatment-resistant from the onset of schizophrenia [16, 17], and another retrospective study showed that delayed clozapine initiation (the time from two failed trials to clozapine initiation) and the duration of illness (the time from onset to clozapine initiation) are significantly positively correlated [18]. Thus, similar results would be obtained using either definition of delayed clozapine initiation.

This study has several limitations. First, this retrospective cohort study relied on medical records, which may have introduced selection bias and incomplete or inaccurate data. For example, important confounding factors such as medication adherence and baseline symptom severity were missing and difficult to adjust statistically. In addition, information on medication history, such as the types and doses of previous antipsychotics, was also unknown. These confounding factors could potentially impact clinical outcomes and bias results. Second, in analyzing our primary endpoint, the number of rehospitalization events, only a maximum of four confounding factors could be adjusted for due to the small sample size, excluding the variable of the time from schizophrenia diagnosis to clozapine initiation. All-cause clozapine discontinuation had even fewer events, but overfitting in the statistical model was a concern because both endpoints were adjusted using the same covariates [19]. Third, the definition of delayed clozapine initiation adopted in this study differs from the most common method [12]. This definition is the duration of illness at the time of clozapine initiation, and may not be strictly indicative of delay in clozapine initiation. Fourth, there is a lack of a comprehensive assessment. This study focused only on rehospitalization and clozapine discontinuation, and the impact of delay in clozapine initiation on psychiatric rating scales and functional outcomes needs to be examined in the future. Finally, although this was a multicenter study, the overall sample size was small and may have limited the generalizability of our results.

## Conclusion

Our results reveal that a longer duration of illness at the time of clozapine initiation, that is, delay in clozapine initiation, increases the risk of psychiatric rehospitalization. Clozapine should be initiated promptly in patients with TRS to improve treatment response and prevent psychiatric rehospitalization in the long term. Further prospective studies with appropriate consideration of confounding factors and large sample sizes are needed to strengthen the evidence.

## Data Availability

The datasets used and/or analyzed during the current study are available from the corresponding author (hatanomasakazu@yahoo.co.jp) on reasonable request.

## Abbreviations

CI: Confidence interval
HR: Hazard ratios
TRS: Treatment-resistant schizophrenia
